# Autophagy Plays Multiple Roles in the Soft-Tissue Healing and Osseointegration in Dental Implant Surgery—A Narrative Review

**DOI:** 10.3390/ma15176041

**Published:** 2022-09-01

**Authors:** Alexandra Ripszky Totan, Marina Melescanu Imre, Simona Parvu, Daniela Meghea, Radu Radulescu, Dan Sebastian Alexandru Enasescu, Mihai Radu Moisa, Silviu Mirel Pituru

**Affiliations:** 1 Department of Biochemistry, Faculty of Dental Medicine, “Carol Davila” University of Medicine and Pharmacy, 020021 Bucharest, Romania; 2 Department of Complete Denture, Faculty of Dental Medicine, “Carol Davila” University of Medicine and Pharmacy, 020021 Bucharest, Romania; 3 Department of Complementary Sciences, Hygiene and Medical Ecology Discipline, “Carol Davila” University of Medicine and Pharmacy, 020021 Bucharest, Romania; 4 Department of Professional Organization and Medical Legislation-Malpractice, “Carol Davila” University of Medicine and Pharmacy, 020021 Bucharest, Romania

**Keywords:** autophagy, osseointegration, dental implant, osteoimmunity, wound healing

## Abstract

Dental endo-osseous implants have become a widely used treatment for replacing missing teeth. Dental implants are placed into a surgically created osteotomy in alveolar bone, the healing of the soft tissue lesion and the osseointegration of the implant being key elements to long-term success. Autophagy is considered the major intracellular degradation system, playing important roles in various cellular processes involved in dental implant integration. The aim of this review is an exploration of autophagy roles in the main cell types involved in the healing and remodeling of soft tissue lesions and implant osseointegration, post-implant surgery. We have focused on the autophagy pathway in macrophages, endothelial cells; osteoclasts, osteoblasts; fibroblasts, myofibroblasts and keratinocytes. In macrophages, autophagy modulates innate and adaptive immune responses playing a key role in osteo-immunity. Autophagy induction in endothelial cells promotes apoptosis resistance, cell survival, and protection against oxidative stress damage. The autophagic machinery is also involved in transporting stromal vesicles containing mineralization-related factors to the extracellular matrix and regulating osteoblasts’ functions. Alveolar bone remodeling is achieved by immune cells differentiation into osteoclasts; autophagy plays an important and active role in this process. Autophagy downregulation in fibroblasts induces apoptosis, leading to better wound healing by improving excessive deposition of extracellular matrix and inhibiting fibrosis progression. Autophagy seems to be a dual actor on the scene of dental implant surgery, imposing further research in order to completely reveal its positive features which may be essential for clinical efficacy.

## 1. Introduction

Modern implantology was made possible thanks to Brånemark’s studies in the 1960s in Sweden. He was the first to propose the concept of osseointegration of a metallic biomaterial implanted in bone [1].

Dental implants are inert, alloplastic materials embedded in the maxilla and/or mandible for the management of tooth loss and to aid replacement of lost orofacial structures as a result of trauma, neoplasia, and congenital defects [1,2].

The most used type of implant is the root-form implant. This type of implant consists of three main components: (1) fixture, (2) abutment, and (3) prosthesis [2,3]. (1) The fixture is represented by a cylinder-shaped metal post, which is surgically embedded into the bone, simulating the root of a tooth. (2) The abutment is attached to the fixture by an abutment screw, which raises from the bone to above the mucosal surface [2,3]. (3) Finally, the prosthesis is cemented to the implant or attached with a prosthesis screw.

Brånemark et al. described for the first time the process of osseointegration [3]. Their findings initiated a new research field oriented towards the shapes and materials of dental implants. However, for only a few years, research has focused on the osteoinductive potential of dental implant surfaces [4]. Implant surface properties, such as coatings, topography, and wettability, play important roles in the soft tissue and osseointegration of the dental implant [5]. The soft tissue and osseointegration are mediated by molecular events triggered by direct interaction between host cells and the implant surface [5].

Given the central roles played by autophagy in sustaining cell survival, proliferation, and differentiation, this complex molecular pathway should be regarded as one of the main actors in the oral post-surgical soft-tissue wound healing and implant osseointegration scene.

This review aims to explore the multiple roles played by autophagy in the main cell types involved in the healing and evolution of the oral soft-tissue lesion and implant osseointegration, respectively: macrophages (MFs); endothelial cells (ECs); osteoclasts (OCs); osteoblasts (OBs); fibroblasts (FBs); myofibroblasts (MFBs); keratinocytes (KCs) (Table 1).

## 2. Dental Implant Surgery Lesion Healing and Implant Osseointegration

Dental endo-osseous implants are placed into a surgically created osteotomy in the alveolar bone. Generally, alveolar bone is accessed via a surgical incision in the mucosa.

Following implant surgery, it is important to focus on two aspects that take place simultaneously: the soft tissue wound healing and the osseointegration of the implant.

The transmucosal attachment is formed after the adaptation of the mucosal wound edges to the transmucosal part of the implant. Unlike tooth soft-tissue attachment formed simultaneously with periodontium, the soft-tissue attachment around the dental implant develops after surgical intervention [39].

In normal physiological conditions, soft-tissue wound healing is a highly ordered biological process. Generally, this complex process includes the following phases: (1) hemostasis and inflammation, (2) proliferative phase, and (3) remodeling phase [39].

**Figure 1:**
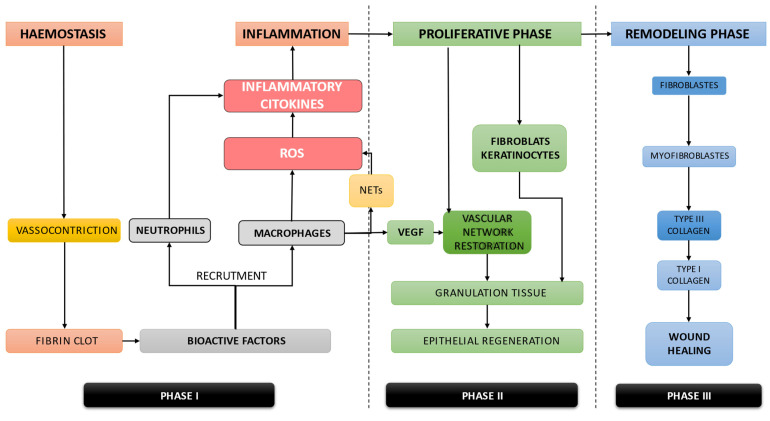
The oral soft-tissue wound healing, a highly ordered biological process, comprises the following overlapping phases: (1) hemostasis and inflammation, (2) proliferative phase, and (3) remodeling phase.

1. Wounding is immediately followed by blood vessel constriction and coagulation cascade triggering clot generation. Fibrin clots have hemostatic effects and also expose a temporary extracellular matrix (ECM) that encourages cell migration [39] (Figure 1). Fibrin clots also release chemical signals in order to recruit the inflammatory cells to the wound scene. The neutrophils that reach the injury site remove necrotic tissue. The recruited monocytes differentiate into MFs that act as phagocytes and initiate the secretion of inflammatory cytokines. These cytokines will trigger local immune reactions [39] (Figure 1).
2. Following the inflammatory phase, the next step of the wound healing process is the proliferative phase, which includes: (1) vascular network remodeling, granulation tissues formation, and epithelial regeneration; (2) MFs, important sources of growth factors, release of vascular endothelial growth factor (VEGF) to initiate vascular remodeling by ECs activation; (3) FBs migrate to the injury site and synthesize collagen, fibronectin, consequently initiating the ECM organization. KCs proliferation and their migration from the edges of the wound to the wound center are essential for wound closure (re-epithelialization) [39,40,41,42] (Figure 1).
3. Remodeling is the final phase of wound healing. This step is characterized by type III collagen replacement with type I collagen within the granulation tissue. FBs and the FBs derived MFBs play key roles in further wound sealing. Additionally, granulation tissue degradation and blood vessel degeneration generate the avascular and acellular mature wound [39] (Figure 1).

Osseointegration depends on the formation of a direct implant-bone interface and represents a key element in the successful establishment of the dental implant [28,43]. Over time, several implant modifications have been designed in order to improve osseointegration. These modifications have included implant shape changes, implant surface feature improvements, and growth factor or other biological stimuli addition in order to stimulate osteogenic differentiation and osteoblast activity [28,43].

## 3. Autophagy Mechanism

There are three essential forms of autophagy called macroautophagy, microautophagy, and chaperone-mediated autophagy (CMA). The main difference between them represents their physiological functions and pattern of delivery [44].

Macroautophagy (referred to hereafter as autophagy) can be depicted as the process that involves the formation of multiple membrane structures, beginning with the phagophore to the formation of the autophagosome, and finally, to the autolysosome [44]. Autophagy, a very complex molecular process, is dependent primarily on the ATG (autophagy-related) family of proteins [45].

**Figure 2:**
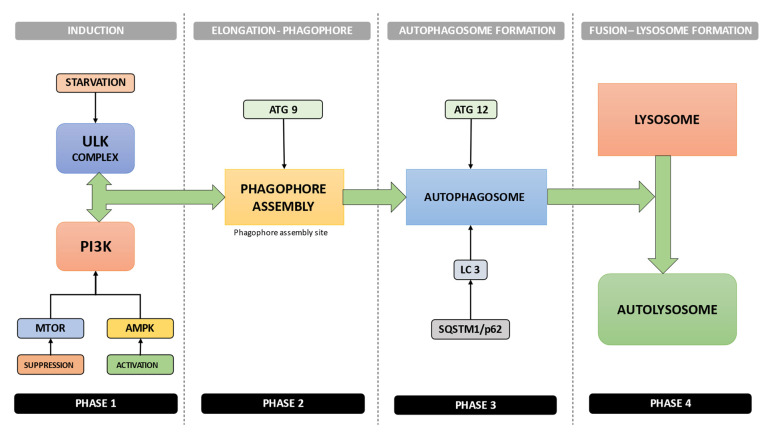
Molecular events sequence in autophagy: (1) activation of the ULK complex by signals such as starvation; (2) phagophore assembly at the phagophore assembly site; (3) phagophore elongation to the autophagosome; (4) the autophagosome fuses with a lysosome, resulting the formation of autolysosome.

The molecular events sequence in autophagy is, in short, as follows (Figure 2):

1. Activation of the ULK complex by signals such as starvation, which will subsequently bind to the PtdIns3K complex following mTOR suppression or AMPK activation [44] (Figure 2);
2. After the induction, the combined, orchestrated action of ULK complex, PtdIns3K complex, and ATG9 complex will trigger the phagophore assembly at the phagophore assembly site [44] (Figure 2);
3. The conjugation systems ATG12 and LC3 are key factors in the regulation of the phagophore elongation to the autophagosome. Autophagy is suppressed by mTOR, the major autophagy inhibitory factor, as a response to abundant nutrient conditions. Class I PI3K and AKT signaling mediates this suppressive action [44] (Figure 2);
4. The receptor protein SQSTM1/p62 (sequestosome 1) will subsequently interact with both LC3 and ubiquitin chains [44] (Figure 2);
5. Subsequently, the autophagosome will fuse with a lysosome, resulting in the formation of the autolysosome. The autophagosome constituents placed inside the autolysosome will be hydrolytically degraded. The engulfed SQSTM1 complex will be degraded inside the autolysosome, which emphasizes SQSTM1’s role as an autophagy flux marker [44] (Figure 2).

## 4. Autophagy in the Main Cellular Types Involved in Dental Implant Surgery Lesion Healing and Implant Osseointegration

### 4.1. Autophagy in Macrophages (MFs)

In oral post-surgical lesions, neutrophils are key cells that enter the wound in order to phagocytose and eliminate contaminating microorganisms. These cells’ recruitment is triggered by chemical signals released by platelets [45,46,47]. During the inflammatory phase (Figure 2), the neutrophils and monocytes are recruited to the wound site [46,47,48]. The proinflammatory and antimicrobial effects of neutrophils are primarily based on phagocytosis, degranulation, reactive oxygen species (ROS) generation, and neutrophil extracellular traps (NETs) release [46,47,48,49]. Several studies have highlighted the close connection between autophagy and the specific biological functions of neutrophils [39,50,51,52]. Ullah et al. study revealed that the autophagy inhibition in neutrophils has seriously reduced the phagocytic function. Moreover, Bhattacharya’s results have pointed out that the neutrophils from mice with mutations in specific autophagy-related genes (*atg5/7*) have shown intensely decreased levels of degranulation and ROS formation [50].

However, from all cell types involved in the innate immune response to an implant, MFs should be regarded as the main actors on the stage of implant surgery wound healing [47,49]. Oral tissue macrophages, differentiated from circulating monocytes, migrate into the wound and play main roles in the immune regulation and the release of growth factors needed to induce the proliferation and migration of connective tissue cells of the periodontium [47,49].

MFs are heterogeneous cells, able to differentiate into different phenotypes depending on the microenvironment-specific features: (1) phenotype M1 are MFs that promote the release of the inflammatory factors release during the early inflammatory phase and kill pathogens; (2) phenotype M2 MFs suppress the immune reactions during the late inflammatory phase, consequently inducing and sustaining tissue repair and wound healing [53]. During the stages of the wound (including the implant surgery ones) healing process, MFs are the main sources of growth factors. They release the vascular endothelial growth factor (VEGF) in order to stimulate ECs activity for vascular remodeling [39].

Zhu et al. have shown that both in vivo and in vitro autophagy inhibition with 3-methyladenine increased MFs phagocytic activity [54]. It also has been outlined that MFs autophagy downregulation promoted their polarization towards the M1 phenotype. Moreover, autophagy initiation stimulated the polarization towards the M2 phenotype, to attenuate the inflammatory reactions and sustain tissue repair [7,55,56]; however, the precise molecular mechanism that controls autophagy-mediated M1-M2 conversion is still unclear. Consequently, further studies are required for a deeper understanding of the complex relationships that connect autophagy and MFs functions.

The multifunctional pathway, autophagy, together with as yet unexplored functions, plays key roles in essential immunity molecular events, such as MFs differentiation and pathogen elimination [6,57]. The autophagy immunomodulatory roles have been highlighted in both innate and adaptive immune responses, illustrating autophagy as one of the main regulators and allies in implant surgery wound healing and osseointegration [6,57]. Young et al. have demonstrated that autophagy inhibition, pharmacological intervention, or *atg* gene deletion have induced IL-1b secretion of MFs, suggesting autophagy limits the inflammatory response of MFs [6]. This MFs inflammatory response limitation might be very important in the last stages of implant surgery wound healing and in ensuring the implant osseointegration.

It has been demonstrated the key role of autophagy in MFs polarization and inflammatory response regulation [6,58], revealing the possible important contribution of this molecular pathway in implant surgery wound healing and, also, in the osseointegration of the implant. During M1, MFs polarization autophagy is induced by toll-like receptor 4 (TLR4) signaling [6,58]. In addition, further research has led to the conclusion that autophagy played an immunosuppressive role in the MFs-induced inflammatory response [6,59,60,61]. For instance, *Atg16L1* and *Atg5*- deficiency triggered the direct polarization of phenotype M2 MFs toward the M1 phenotype, with increased pro-inflammatory cytokines secretion [6]. Moreover, it has been reported that inflammatory signals caused mitochondrial damage, and, consequently, induced increased ROS release in MFs [6]. The released ROS interact with the NF-kB signaling pathway and then activate the NLRP3 inflammasome. NLRP3 inflammasome activation induces IL-1b and IL -18secretion, triggering the inflammatory cascade initiation [6]. However, if, during this process, the damaged mitochondria are efficiently scavenged by autophagy via p62 and LC3 collaboration, the inflammatory cascade may be interrupted. p62 selectively recognizes damaged mitochondria through its UBA domain; after the recognition process, p62 interacts with LC3, ensuring the lysosomal degradation of damaged mitochondria and, consequently, the interruption of the inflammatory cascade [6].

MFs autophagy is also involved in direct or indirect stimulation of pathogen clearance [62,63], revealing once again the importance of this pathway in the implant surgery context, especially in the first stage of wound healing. MFs can recognize the pathogen molecular patterns also via pattern-recognition receptors (PRRs). The interactions with PRRs induce autophagy and further, initiate the innate immune responses [62,63]. There are two common PRRs (Toll-like receptors (TLRs) and Nod-like receptors). TLRs are able to induce autophagy by activating the TGF-β-activated kinase–AMPK axis [62,63].

Lately, with the progress of immunology and a deeper understanding of the immune system—bone remodeling relationships, the new notion of “Osteoimmunity” is emerging more and more. Osteoimmunity should be considered an important player in the post-surgery evolution of the dental implant because its osseointegration is orchestrated by inflammatory processes [64,65].

Optimizing implant surface properties to steer the immune response to an implant has been an area of increasing research focus [65].

MFs are also important players in “osteoimmunity” [65]. The MFs are one of the main actors of innate immunity, playing key roles in immune regulation [64,65].

After implant insertion, the host soft-tissue damage leads to blood extravasation, triggering the activation of the host’s immune response. After implantation, MFs are the first immune cells to contact the implant [65]. MFs can secrete cytokines and molecular mediators, including proinflammatory cytokines (TNFα), anti-inflammatory mediators (IL-10), and growth factors (such as transforming growth factor beta—TGFβ) [65]. These cytokines and molecular mediators are involved in the host immune response regulation, deeply affecting the interaction between the host cells and the implant at the material–cell interface [65]. The immune system, especially through MFs, plays an essential role in post-implant surgery bone regeneration and implant osseointegration [65].

It has been pointed out that the MFs-induced inflammation triggered OCs-genesis and bone loss; however, the conversion of the proinflammatory phenotype M1 toward the anti-inflammatory M2 phenotype seemed to initiate and improve bone repair [66]; therefore, the autophagy-mediated M1 toward M2 conversion should be considered crucial for bone regeneration and implant osseointegration. For instance, it has been shown that the nanomaterials-derived autophagy induction has triggered MFs conversion toward M2 phenotype, consequently improving osteogenesis [8]. Experimental data led researchers to the conclusion that IL-17 has initiated osteogenesis; however, excessive IL-17 production has resulted in increased RANKL secretion and OCs-genesis [9,10]; therefore, IL-17 is still considered unfavorable for bone regeneration, and consequently, for the implant osseointegration. Moreover, it has been revealed that autophagy-induced conversion of Th1 to Th2 cells might initiate the M1 MFs polarization to the M2 phenotype [67,68], an essential part of bone regeneration [6]; therefore, all these findings suggest that autophagy-mediated immunomodulation of T cells prepares a microenvironment that can sustain bone repair.

**Figure 3:**
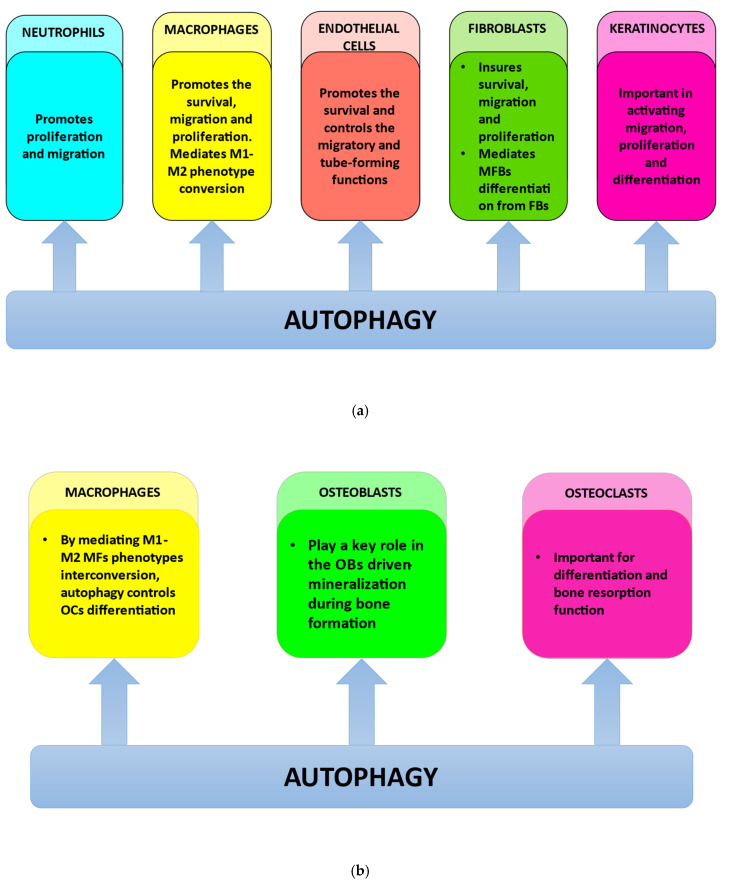
(**a**): Autophagy roles in the main cellular types involved in oral soft tissue healing post-implant surgery: (1) promotes neutrophil proliferation and migration; (2) ensures the survival, migration, and proliferation of macrophages (MFs); mediates M1-M2 phenotype conversion of MFs; (3) sustains the survival of endothelial cells (ECs) and controls their migratory and tube-forming functions; (4) ensures the survival, migration, and proliferation of fibroblasts (FBs); mediates myofibroblasts (MFBs) differentiation from FBs; (5) plays important roles in activating migration, proliferation and differentiation of keratinocytes (KCs). (**b**): Autophagy roles in the main cellular types involved in the osseointegration of the dental implant: (1) by mediating M1-M2 MFs phenotypes interconversion, autophagy controls osteoclasts (OCs) differentiation; (2) plays a key role in the osteoblast (OBs) driven-mineralization during bone formation; (3) is important for the differentiation and the bone resorption function of (OCs).

**Figure 4:**
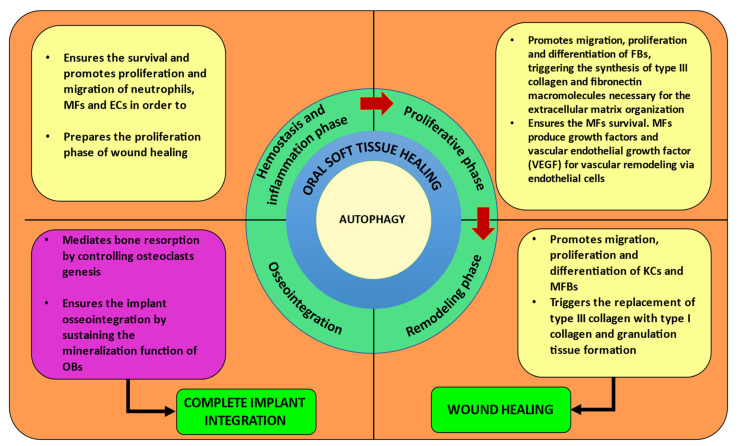
An overview of autophagy multiple roles in the oral soft-tissue healing phases and osseointegration in dental implant surgery.

All these findings highlight the idea that autophagy should be regarded as a potential immunomodulation target in therapeutic strategies for inflammatory disorders accompanied by bone loss, such as periodontitis [11,12], and also for improving osseointegration and preventing dental implant rejections (Figure 3a,b and Figure 4).

### 4.2. Autophagy in Endothelial Cells (ECs)

The ECs form a single-cell layer of the endothelium, playing essential roles in cardiovascular homeostasis by regulating blood fluidity, vascular tone and permeability, monocyte adhesion, platelet aggregation, fibrinolysis, and angiogenesis [13]. ECs, as the inner lining of all sub-vascular compartments, are responsible for supplying nutrients and oxygen to the parenchymal tissue [69]. ECs are sensitive to different stimuli, including low oxygen levels, nutrient availability, oxidative stress, and unfolded proteins. Hypoxia and nutrient deprivation represent common stressful features of the vascular microenvironment that may activate ECs [20]. In such cases, after vascular reperfusion and restoration of physiological levels of oxygen and nutrients, ECs may return to the quiescent state [20].

During the proliferative phase of the oral wound healing process, the platelets and local MFs populations secrete growth factors, including the endothelial growth factor (VEGF), in order to initiate angiogenesis [47,70]. The needed new blood vessels can form from pre-existing capillaries by the endothelial precursor cell’s proliferation and differentiation [47,70].

Intrinsic ECs autophagy modulates the response of these cells to various metabolic stressors and has a fundamental role in nitric oxide production, angiogenesis, thrombosis, and hemostasis [14]. ECs autophagy stimulation induces apoptosis resistance, ensuring cell survival, and also protects ECs against oxidative damage [16,17]. Moreover, Zha et al. have shown that Forkhead Box Protein O3 (FOXO3a) improved the endothelial progenitor cells (EPCs) functions by activating autophagy [18]. Recent studies led to the conclusion that hypoxia-induced autophagy should be regarded as a protective molecular mechanism for ECs and also a key element in inducing angiogenesis, which is an important step during implant surgery wound healing [19,39,71]. Jeong et al. revealed that autophagy downregulation induced by *atg5* expression silencing or by treatment with autophagy inhibitors triggered the inhibition of ECs migratory and tube-forming functions, outlining the indirect but important role of ECs autophagy in angiogenesis [72]. Moreover, Chandel et al. studies revealed for the first time that the protein tyrosine phosphatase PTP-PEST (also known as PTPN12) mediates hypoxia-induced AMPK activation and ECs autophagy in order to promote angiogenesis. The authors demonstrated that PTP-PEST knockdown had an inhibitory effect on ECs migration and capillary tube formation, illustrating that autophagy was a central element in angiogenesis orchestration [73].

Several findings have revealed interesting similarities between ECs and MFs [74,75,76]. Generally, ECs are not regarded as classic immune cells; however, they express a variety of innate immune receptors such as NOD-like receptors (NLRs) and Toll-like receptors (TLRs). These receptors activate intracellular inflammatory pathways mediated by nuclear factor kappa B (NF-κB) and the mitogen-activated protein kinases [20]. Interestingly, MFs and ECs have similar evolutionarily conserved mechanisms of defense, such as autophagy and phagocytosis [20,43]. MFs are phagocytes highly specialized in the detection and removal of apoptotic cells, cell debris, and pathogens. In turn, ECs are able to adopt MFs-like functions (that may include phagocytosis, autophagy, and the innate immune response) in order to protect the underlying tissue against blood-generated toxins and pathogens [20,77]; however, the precise mechanisms of ECs MFs-like function are still a mystery [20,77]. Recently, the importance of ECs autophagy, especially in the context of oral post-implant surgery wound healing, has been increasingly recognized; however, ECs autophagy remains a poorly explored field, but surely full of promises, particularly regarding oral implantology (Figure 3a).

### 4.3. Autophagy in Osteoclasts (OCs)

OCs, giant multinucleated cells, are responsible for bone resorption, made by polarized secretion of protons and proteolytic enzymes, in a sealed bone area named resorption lacunae [78]. OCs play essential roles in bone homeostasis.

Rapid and efficient osseointegration represents the key event leading to implant success. Regarding bone remodeling during dental implant osseointegration, OCs activity together with osteogenic cell differentiation on the implant surface is extremely important. In order to achieve an effective peri-implant bone formation and implant osseointegration, the surface of the implant needs to be able to promote osteogenesis and, at the same time, to disfavor bone resorption, via limiting osteoclastic differentiation and reducing OCs activity [79]. Maintaining the balance between OBs and OCs activities represents the key to successful implant osseointegration and prevention of implant rejection.

Osteoclastic differentiation depends on the activation of the RANK receptor, expressed on OCs precursors. Pre-OCs RANK activity is controlled by the relative levels of RANKL and osteoprotegerin (OPG) produced by OBs [80].

Several studies have shown the connection between osteoclast-genesis and the OBs-derived receptor activator of nuclear factor kappa B ligand (RANKL) [81,82], suggesting the existence of a relationship between osteoclast-genesis and osteogenesis [83]. Simonet studies revealed that OCs-precursors RANK bind OBs-derived RANKL, triggering OCs differentiation; however, it also has been shown that OBs produce osteoprotegerin (OPG), a decoy RANKL receptor, in order to block osteoclast-genesis [84,85]. Moreover, osteoclast-genesis and osteogenesis can also be regulated by other OBs-derived factors [86]. For instance, OBs represent one of the main sources of MFs colony-stimulating factor (M-CSF), which is very important for OCs differentiation [78,87,88,89], highlighting the importance of MFs-OBs-OCs interrelations. MFs and other innate immune system cells are OCs precursors [90] and also play important roles in mediating osteoclast-genesis. M1 MFs induces osteoclast-genesis by releasing cytokines such as: IL-1a/b [91,92], IL-6 [93,94], and TNF-a [95,96]. M2 MFs reduce OCs differentiation via IL-10 and TGF-b secretion [53,54,97,98].

Moreover, osteoclast-genesis can also be inhibited by OBs-derived semaphorin 3A (Sema3A) and Wnt16 via interrupting the RANKL-RANK connection [99,100]; however, it has been demonstrated that OBs Wnt5 activated OCs differentiation by stimulating RANK expression in OCs-precursors [101,102].

Consequently, it can be concluded that the balance between OBs and OCs may decide the fate of bone regeneration and, possibly, of implant osseointegration. An important role in maintaining this OBs-OCs delicate balance, illustrated by the RANK/OPG ratio, belongs to autophagy [103,104]. In the dental implant surgery context, the RANK/OPG ratio can be regulated by the implant surface, outlining the fact that the implant surface properties play subtle but very important roles in achieving successful osseointegration [80].

Autophagy plays an important role in OCs differentiation [103,104]. Chung et al. highlighted the role of Beclin-1 in OCs differentiation. They have shown that bone marrow MFs Beclin-1 was involved in RANKL-induced osteoclast-genesis by increasing ROS production and inducing the master gene NFATc1expression, but in an autophagy-independent manner [21]. The study of DeSelm et al. revealed that autophagy-related proteins Atg5, Atg7, Atg4B, and LC3 illustrated an autophagy-independent function in the OCs ruffled border generation, secretory activity, and bone resorption [22]. Atg5 is needed for LC3-II targeting the ruffled border. LC3-II presence may induce the fusion with secretory lysosomes required for bone resorption [22]. Further, Chung et al. research showed that OCs functions required the LC3-I to LC3-II conversion, but without increasing the autophagic flux [21,23]. These authors demonstrated that LC3 knockdown had no effects on TRAP-positive multinucleated cell differentiation but suppressed the bone-resorbing capacity of OCs [21,23].

Taking all these together, it can be concluded that autophagy or at least some autophagic proteins should be regarded as the main actors in the OCs differentiation and functions. Consequently, in the case of OCs, the autophagic pathway and/or autophagic proteins activities should be considered important further research targets with possible negative effects on the satisfactory evolution of the dental implant osseointegration (Figure 3b and Figure 4).

### 4.4. Autophagy in Osteoblasts (OBs)

OBs are specialized, mesenchyme-derived cells responsible for bone formation. These cells are considered professional mineralizing cells [105]. During bone formation, some OBs are entrapped in their own matrix. Some of them differentiate into osteocytes, but most of them will initiate apoptosis [105].

The dental implant surgery success depends on the efficient integration of the implanted biomaterial into bone tissue. It is important to mention the fact that dental implant osseointegration is sustained by the adhesion, proliferation, and differentiation of osteoblasts OBs [80,105]. The adhesion process is followed by the assembly of a mineralized matrix directly on the biomaterial surface [80,105]; however, 5–11% of dental implants are estimated to fail within 10–15 years, due to improper osseointegration and peri-implant bone loss. These implants will be removed [80,105]. So, in order to improve the success rate of dental implant surgery, it is crucial to clarify the molecular landscape of dental implant osseointegration and to elucidate the molecular mechanism of peri-implant bone loss.

Both sRANKL and OPG are synthetized by OBs. sRANKL is exposed on OBs surface and activates OCs. RANKL decoy receptor OPG (or the osteoclast genesis inhibitory factor) counteracts RANKL biological functions by preventing its interaction with its receptor (RANK) [21,23]. Consequently, alterations of the RANKL/OPG balance are critical for the new bone formation [21,23] and, finally, for the implant osseointegration process.

Regarding dental implant osseointegration, the cell–cell and, especially, biomaterial–cell communications are vital, mainly in the early post-surgery stage [106]. Trindade et al. highlighted that the entire osseointegration process is controlled by inflammatory signals [64]. Further studies of osteoimmunology revealed the complex interrelations between immune cells and bone cells [107]. More precisely, the immune responses control the initiation and the result of bone remodeling, but at the same time, bone cells mediate the polarization and, consequently, the functions of immune cells [107]. The complex bone cell–immune cell interactions are based on the action of multiple factors such as cytokines, receptors, and signaling pathways [107].

Autophagy is a key player in the OCs and OBs differentiation [108,109] but also a main participant in the immune cell polarization process; therefore, autophagy’s involvement in bone cells’ differentiation and in immune cells’ polarization suggests a subtle, complex but critical role in osteoimmunology [15,24,25,26] (Figure 3b).

The main finding of Ting Zhang’s study is that autophagy can effectively regulate the osseointegration of implants into the osteoimmune microenvironment [65].

Interesting findings revealed that the pro-inflammatory cytokine IL- 6 induced osteogenesis via the oncostatin M (OSM)-STAT3 signaling pathway, suggesting that at a certain level, the inflammatory responses could initiate OBs differentiation [15,24,25,26]. These results are in accordance with the findings showing that the early-stage inflammatory responses along with MFs infiltration should be considered crucial in post-surgery bone lesion healing [15,24,25,26]; however, these inflammatory responses will be gradually attenuated as M1 MFs are converted to M2. Normally this M1-M2 conversion takes place along with bone repair and improves the quality of the new-formed bone [15,24,25,26]. The M2 MFs-derived factors BMP2 and TGF-b promote OBs differentiation and functions, including mineralization [15,24,25,26].

Li et al. outlined that autophagy plays an important role in the OBs driven mineralization during bone formation. Autophagy may be regarded as a carrier that transports mineralization-related factors, wrapped in stromal vesicles, to the extracellular matrix [110]. Studies using OBs cell lines demonstrated that the autophagic flux was increased during OBs differentiation and then during the mineralization process [110], highlighting the important role played by autophagy in these two processes, which are crucial for bone lesion healing and dental implant osseointegration.

The key role of autophagy on the OBs functions scene has also been confirmed by analyzing the consequences triggered by autophagic flux inhibition [110]. The results illustrated a decrease in the size and number of alkaline phosphatase-positive cells, leading to a significant downregulation of the mineralization process [110]. As the autophagy inhibitory treatments had no effects during the early stage of OBs differentiation, the authors outlined the conclusion that autophagy was required for the final steps of OBs differentiation [110]. In conclusion, besides its subtle and indirect role played in the final stage of differentiation, OBs autophagy seems to be directly involved in the OBs-driven mineralization process via minerals secretion in the extracellular space.

Osteocytes (OSTs) represent the final differentiated state of the OBs and are embedded within the mineralized bone matrix. Due to their location and long life span, OCs are submitted to stressful conditions. As a result, it is expected that their survival is highly dependent on the autophagic machinery. Both OBs and OSTs are mechanosensitive cells [111]. Bone adaptative responses to loading are mediated by OBs and OSTs, both able to perceive and transform mechanical forces into a molecular signaling cascade, which will induce biochemical and structural changes [111]. Klein-Nulend et al. demonstrated that in mammalian cells, autophagy is highly sensitive to mechanical pressure changes [111]. These authors also outlined that the mechanical activation of autophagy was transient and mTOR-independent [111].

Most of the few studies that focus on the multifaced role of autophagy in dental implant osseointegration are in vitro studies; however, these implants are clinically placed in the oral cavity, facing a really challenging microenvironment; consequently, further investigation is impetuously needed in order to understand the complex connection between autophagy and implant osseointegration in vivo (Figure 3b and Figure 4).

### 4.5. Autophagy in Fibroblasts (FBs) and Myofibroblasts (MFBs)

Compared to cutaneous wounds, oral wound healing is characterized by an accelerated rate [112]. Additionally, Torres et al. revealed that wound healing in gingiva and oral mucosa is more efficient and faster compared with skin or other mucosal tissues [113]. The molecular mechanisms that generate these differences are still unclear; however, it has been established that FBs are essential protagonists in the wound healing context [113,114].

The wound repair process involves multiple cell types, of which FBs play essential roles since they, alongside with ECs, orchestrate the synthesis of the replacement extracellular matrix (ECM) [47,105,115,116,117]. FBs are the main actors in both the proliferative and remodeling phases of wound healing (Figure 2) [27,117]. During oral surgery wound healing, the inflammatory cells synthesize and secret several mediators that activate and recruit resident fibroblasts at the wound margin [118]. Fibroblasts migrate to the wound site, where they synthesize collagen and fibronectin that support the ECM restoration. FBs are also involved in granulation tissues formation [27,47,118].

Peri-implant mucosal connective attachment tissue has some similar histological and clinical features to that of the teeth [119]; however, the peri-implant mucosal connective attachment tissue presents significant differences regarding matrix fibers orientation and cellular composition. The dental implant surrounding connective tissue is in direct contact with the implant surface and contains a dense network of collagen fibers originating from the alveolar bone crest periosteum and extending to the mucosal margin [119]. These collagen fibers are orientated in parallel to the implant/abutment surface [119], in contrast to the attachment of connective tissue to the tooth. In the case of the tooth, the collagen fibers are inserted perpendicularly into the root cementum [119]. In a human study, Tomasi et al. reported that the peri-implant mucosal seal was completed in 8 weeks of healing [119]. In an animal study, Moon et al. revealed the presence of a large number of FBs within the peri-implant attachment soft tissue close to the implant surface (Moon et al. 1999) [120]. The long axis of these FBs was orientated in parallel to the adjacent collagen fibers and to the implant surface [120]. The authors outlined the conclusion that the fibroblast-rich barrier tissue, located immediately next to the implant surface, plays an essential role in inducing and maintaining an adequate seal against external factors [120]. FBs migration and differentiation capabilities represent the main elements for the FBs-rich barrier tissue organization. Moreover, Migneault et al. revealed that autophagy is deeply involved in orchestrating the FBs differentiation [29].

Zhou et al. study illustrated that the transcription factor EB had an important role in mediating the autophagy pathway, consequently ensuring FBs survival [27]. Additionally, it has been pointed out that remifentanil, a synthetic, short-acting, opioid analgesic drug, inhibited oxidative stress (OS) induced apoptosis in skin fibroblasts via autophagy activation [121]. In a rat model of wound healing, Asai et al. study of the spatio-temporal changes in LC3-positive dots in FBs and MFBs, illustrated a notable increase in the number of LC3-positive dots during the late proliferative phase [122]. The authors reported that the number of the LC3-positive dots was higher at the injury edge compared with the center of the wound, leading to the idea that the wound edge FBs were in the differentiation phase; highlighting the important role played by autophagy on the FBs differentiation scene [122]; however, Cao et al. revealed in their study that autophagy was not involved in gingival wound healing, leading to fewer myofibroblast differentiation [28]. The authors have also highlighted that FBs autophagy downregulation induced the apoptotic pathway [28]. On the other hand, it has been shown that inflammation activated autophagy. Conclusively, autophagy may play dual parts in the wound healing scene, triggering different clinical consequences depending on the cell type within which it takes place [28,122].

Gingival FBs are the main players in maintaining periodontal tissue homeostasis via active migration and proliferation [113,114]. It has been demonstrated that immediately after implant surgery, the wound-healing ability of the gingival tissue can be maintained even in a hypoglycemic microenvironment [113,114]. It has been outlined that FBs’ capacity to migrate and proliferate is decisive in terms of oral tissue wound healing [113,114,123]. FBs use the glycolytic pathway to sustain cell migration and proliferation [30]. In the past 20 years, it has been outlined the important roles played by the glucose metabolic pathways in the FBs proliferation stage. These molecular pathways represent sources of energy and supply of metabolites, vital to sustain the biosynthesis of nucleic acids and membrane lipids [124]. In the cases of post-implant-surgery lesions, where the existing vasculature is destroyed, the cells face stressful microenvironments characterized by low glucose levels [30]. Glucose starvation triggers an increased ROS formation [125,126]. The enhanced ROS levels can activate the LKB1-AMPK signaling pathway, and consequently, induce autophagy [125,126]. Presently, the precise role of the LKB1-AMPK signaling pathway and autophagy in oral soft tissue wound healing, especially under hypoglycemic conditions, is still unclear [125,126]. The studies of Scherz-Shouval et al. have demonstrated that increased levels of ROS could induce autophagy via the LKB1-AMPK signaling pathway in order to prevent cell damage, but without activating apoptosis [127].

Autophagy is regarded as a regulatory mechanism of the cell, responsible for removing unnecessary and dysfunctional cellular components or proteins, ensuring in this way their systematic degradation and recycling [32,33]. It also has been highlighted that the autophagic pathway represents an important adaptive response to stress. Actually, autophagy has a central role in maintaining cellular energy levels and sustaining cell survival. Several studies reported that in periodontal tissues, autophagy could be induced by inflammation [30,32,33]. The authors have studied both p62 and LC3B as autophagy markers [30,32,33]. Li et al. results illustrated that low glucose levels induced an increased expression of LC3B and p62 [30]. These results outline that FBs autophagy may be induced by low glucose levels alone, independently of inflammation, sustaining in this way the cell survival and preventing cell death. Autophagy and apoptosis are closely interrelated [30], given that it has been shown that both apoptotic cell death and the autophagic machinery might be controlled by the same genes. Moreover, the autophagic signaling pathway is able to regulate apoptotic cell death, while apoptosis may also mediate autophagy [31]. Li et al. results led to the conclusion that low glucose-induced autophagy may inhibit cell death. These results highlighted the key role of autophagy in cell survival and cell death prevention in glucose starvation [30]; however, the landscape of autophagy and apoptosis molecular connections still remains unclear.

It has been shown that the molecular mechanisms of oral soft tissue wound healing are accompanied by reduced inflammatory responses, which can explain the reduced scar formation [128,129,130]. After the granulation tissue has produced enough collagen, the wound contraction can start [118]. Wound contraction and remodeling are actively controlled by myofibroblasts (MFBs) [118]. MFBs are formed from resident local FBs or other progenitor cells. MFBs differentiation is activated by the transforming growth factor-β (TGFβ), and the mechanical forces generated during granulation tissue development [131,132,133]. MFBs express α-smooth muscle actin (αSMA) and are characterized by a strong actin-rich cytoskeleton, enabling them to contract the matrix using integrin receptors [134,135]. Additionally, MFBs represent an important cellular source of type I collagen, vital for the wound healing process. Generally, after the wound is fully covered by epithelium, MFBs apoptosis is initiated [131].

The way the wound repair takes place is related to the FBs phenotype, αSMA expression level, and contraction rate [136]; however, scarless oral soft tissue wound healing still remains a mystery. Since it has been generally accepted that MFBs are the main actors in the physiological reconstruction of connective tissue in surgery-induced lesions, a deeper understanding of their role might be crucial in order to clarify the molecular events that generate the differences between the oral wound healing process and skin healing [131]. Mallat et al. results illustrated that in some cellular contexts, autophagy stimulation could trigger fibrotic disease [137]. Starting from these studies, Vescarelli et al. have explored the possible molecular mechanisms involved in gingival and oral mucosa wound repair [34], particularly focusing on MFBs autophagy. The authors’ findings highlighted an interesting link between autophagy and MFBs differentiation in the oral soft tissues [34]. They have shown that autophagic machinery inhibition significantly decreased MFBs differentiation induced by the TGFβ treatment, giving autophagy a key part in mediating collagen deposition and post-wounding scar formation [34]. Vescarelli results revealed that the early steps of oral wound repair are characterized by decreased αSMA fibers synthesis and collagen deposition, as a consequence of the autophagic pathway downregulation, which probably triggered oral tissue repairing without fibrosis and scar formation [34]. Moreover, Vescarelli’s findings regarding autophagy involvement in MFBs differentiation outlined the role played by the inflammatory responses in the oral wound healing context [34]. More exactly, inflammatory signals that activate MFBs increase active TGFβ release, which in turn might activate autophagy, triggering MFBs’ persistent activation, which can induce excessive scarring [34]. Conclusively, clarifying these molecular mechanisms and their interrelationships should highlight the importance of inflammation management during the early postoperative period in implant surgery and may be helpful in creating new therapeutic strategies (Figure 3a).

### 4.6. Autophagy in Keratinocytes (KCs)

The proliferative phase represents the second step of the wound healing process, after the inflammatory phase, and involves the vascular network restoration, granulation tissue generation, and epithelial regeneration. KCs proliferation and their migration from the injury edges to the center of the wound represent key steps in the epithelial regeneration process [70]. Activated keratinocytes are able to migrate along the previously formed fibrin blood clot into the higher layers of the granulation tissue. This process represents the KCs ‘shuffling’ and illustrates KCs’ capability to migrate competitively over a fibronectin-rich matrix toward the injury center, along a chemotactic gradient generated by immune mediators such as IL-1. The migration process is possible due to the enzymatic loosening of intercellular desmosomes ensured by collagenase and elastase activities [70].

On the oral wound healing scene, the migrating keratinocytes’ interactions with underlying matrix molecules (such as fibrin, fibronectin, and type I collagen) are vital for the restoration of the oral epithelial tissue [47]. The molecular functions of epithelial cell integrins are interrelated with the increased expression and enzymatic activity of the matrix metalloproteinases. These enzymes degrade collagen facilitating the directional migration of keratinocytes [47].

Regarding dental implants, the targets to be achieved are long-term biochemical, biological, and mechanical stability. This stability depends on the quality of the integration in both soft tissue and the underlying supporting bone [138,139]. Peri-implantitis, the destructive soft tissue–implant interface inflammation, is considered one of the main causes of implant failure [140,141]. Over time, Titanium (Ti) and its alloy’s surface properties have been intensively studied, in order to sustain efficient osseointegration, to finally enhance the clinical success rate of implant surgery [41,142,143,144]. The interface between the dental implant and soft tissue represents a key element for obtaining long-term implant stability; however, less attention has been granted to this interface, characterized by a delicate balance [138,145].

Studies in 2D illustrated that dental implant surface properties were important for inducing the attachment and growth of gingival keratinocytes and fibroblasts [57,146,147,148,149]. Conversely, smother surfaces promote a pro-inflammatory polarization of MFs, while rougher surfaces were shown to stimulate MFs’ differentiation towards the anti-inflammatory M2 phenotype [150]. Lackington et al. findings highlighted that the blood–implant interactions, modulated by the implant surface properties, led to variable degrees of fibrin network assembly [57]. This fibrin network represents a protein-based support structure and also a cytokine reservoir, which will then rule the response of surrounding soft tissue cells, especially FBs and KCs [57]. Specifically, the nature of the interactions between these cell types, the fibrin network, and cytokines will have a decisive effect on the migration and proliferation abilities of FBs and KCs, mandatory not only for the epithelium regeneration in the implant surgery wound but also for the soft tissue integration of the dental implant. Lackington et al. results revealed that KCs were particularly sensitive to the Ti implant surface properties. More precisely, Ti implant smoother surfaces and a limited fibrin network created more favorable conditions for KCs attachment and proliferation [57]. In contrast to KCs behavior, rough-surfaced implant–blood interactions stimulated enhanced fibroblast attachment and induced stronger MFs anti-inflammatory responses compared with the smooth-surfaced implant [57]. Several studies demonstrated the central role played by autophagy in activating KCs migration and proliferation [35,37,151,152,153,154], highlighting the importance of autophagy studies in the context of dental implant surgery wound healing (Figure 3a,b).

Recently, Qiang et al. [35] revealed the important roles played by autophagy in KCs proliferation, differentiation, and migration. The authors found that KCs from the wound neo-epidermis of *atg5/7*-knockout mice presented lower proliferation and differentiation rates compared with wild-type mice [35]. More precisely, Qiang et al. have shown that TNF initiated the expression of KCs autophagy genes via NFKB, consequently inducing the autophagic flux. The authors also pointed out that TNF promoted C-C motif chemokine ligand 2 (CCL2) synthesis via the autophagy-AMPK-BRAF-MAPK1/3/ERK-activator protein 1 (AP1) pathway [35]. Qiang et al. results led to the conclusion that the autophagic pathway enables KCs proliferation and migration through CCL2 [35]. The same authors showed that besides indirectly promoting KCs proliferation and migration, KCs autophagy also initiated fibroblast activation via CCL2 [35].

Wang et al. results revealed that deficient autophagy downregulated the PDZ Binding Kinase (PBK) (a member of the mitogen-activated protein kinase (MAPK)) expression in human epidermal keratinocytes [38]. Consequently, the decrease in PBK (a regulator of the cell cycle) expression triggered the inhibition of proliferation in human epidermal KCs. This reduced proliferation can be reversed by activating p38, the downstream signal effect of PBK [38]. Collectively, all these findings highlight the conclusion that autophagy is a positive mediator of KCs proliferation, in part through PBK expression control [38]. Zhang et al. showed that the hypoxic microenvironment generated during the early stage of wounding increased ROS production, which consequently activated the p38 and JNK–MAPK signaling axis, triggering the BNIP3-mediated autophagy upregulation, in order to induce KCs migration [36]. The increased involvement of autophagy in promoting KCs migration and proliferation confer this molecular pathway an important positive role in the stage of dental implant surgery wound healing (Figure 3a).

## 5. Conclusions

During the oral soft tissue wound healing process, at the level of each stage, autophagy plays important and multiple roles. Autophagy ensures the survival, proliferation, and migration of neutrophils, MFs, ECs, KCs, FBs, and MFBs, sustaining their biological functions and, consequently, promoting and mediating wound healing.

Autophagy is also a key molecular pathway involved in immune cell polarization/activation, able to maintain the delicate balance between their anti-infective and anti-inflammatory activities, both crucial for the oral soft tissue wound healing process and implant osseointegration. Consequently, a deeper understanding of all the complex roles played by autophagy during each phase of wound healing is very important in order to elaborate new surgical techniques and treatment strategies to achieve rapid oral soft-tissue wound healing and ensure better dental implant osseointegration. Moreover, decoding the molecular mechanisms that place autophagy on the inflammation scene will draw even more attention to the importance of inflammation management, especially in the early postoperative stage (Figure 3a,b and Figure 4).

The essential part of autophagy in the osteogenesis scene is illustrated by its involvement in osteoblast differentiation and the mineralization process. Autophagy modulatory interventions in both the immune system and bone highlight the crucial roles played in the osteoimmunology landscape. Special consideration should also be given to the blood-implant interactions, the differences between soft and hard tissue’s preferred-implant surface features, and the complex interrelations between cell types adhering to the different parts of the dental implant. Modern fabrication techniques should enable the production of surface-properties gradients in order to achieve optimal implant integration within both soft tissue and the underlying bone.

Until now, targeting the autophagic machinery with small-molecule modulators of specific kinases seems to have become a promising strategy for treating several human diseases, including wounds; however, the duality of autophagy effects in the context of dental implant surgery imposes further research to completely uncover its promising clinical efficacy.

## Figures and Tables

**Table 1:** Summary of autophagy roles in the main types of cells involved in soft-tissue healing and osseointegration in dental implant surgery.

Studied Cell Type	Roles of Autophagy	References
Macrophages (MFs)	1. pathogen elimination mechanism	[6]
	2. antigen presentation and inflammation regulation	[6,7]
	3. IL-1b secretion and, consequently inflammatory response limitation	[6]
	4. MFs polarization	[6,8,9,10]
	5. potential immunomodulation target in regenerative medicine	[11,12,13]
Endothelial cells (ECs)	1. Autophagy is involved in ECs adaptation and survival	[14,15]
	2. Autophagy mediates the ECs response to different metabolic stressors and plays an essential role in nitric oxide generation	[14,15]
	3. Autophagy induction in ECs triggers apoptosis resistance, cell survival and protection against oxidative damage	[16,17]
	4. FOXO3a improves endothelial progenitor cells function via autophagy	[18]
	5. Hypoxia-induced autophagy is an inducer of angiogenesis	[19]
	6. ECs MFs-like function includes phagocytosis and autophagy	[20]
Osteoclasts (OCs)	1. OCs precursor MCP-1-induced differentiation is mediated via MCPIP-induced oxidative stress, Beclin-1 upregulation, and autophagy	[21,22,23]
	2. Regulation of hypoxia-induced osteoclast genesis through theHIF-1alpha/BNIP3 signaling pathway	[21,22,23]
	3. autophagy and/or autophagic proteins may be involved in OCs differentiation and function	[23]
Osteoblasts (OBs)	1. a central part in bone regeneration	[6]
	2. role in the process of osteogenic differentiation	[24,25,26]
	3. important role in the mineralization of OBs during bone formation by secretion of mineral outside the cell	[25]
Fibroblasts (FBs) and Myofibroblasts (MFBs)	1. autophagy to ensure fibroblast survival and functions	[27]
	2. autophagy activation important for triggering fibroblast differentiation	[27]
	3. fibroblasts autophagy downregulation triggers apoptosis	[28,29,30,31]
	4. autophagy in FBs may be induced by extremely low glucose levels without the presence of bacteria cells or inflammation thus preventing cell death and ensuring FBs survival	[30,32,33]
	5. myofibroblast differentiation in periodontal soft tissues is connected with autophagy	[34]
	6. myofibroblast autophagy may mediate collagen deposition and scar formation after wound generation	[34]
Keratinocytes (KCs)	1. autophagy plays a main role in hypoxia-BNIP3 signaling dependent epidermal keratinocyte migration 154	[35,36]
	2. autophagy deficiency inhibits keratinocyte proliferation and differentiation 151	[35]
	3. TNF induces expression of autophagy genes through NFKB in epidermal keratinocytes	[35,36,37,38]
	4. keratinocytes CCL2 activation via autophagy is necessary for keratinocyte migration and proliferation	[35,36,37,38]
